# Matrix metalloprotease-7 is associated with post-COVID-19 persistent lung abnormalities

**DOI:** 10.1183/23120541.00266-2024

**Published:** 2024-12-02

**Authors:** Ivette Buendia-Roldan, Leslie Chavez-Galan, Hiram Aguilar-Duran, Andy Ruiz, Ramcés Falfan-Valencia, Gloria Pérez-Rubio, Annie Pardo, Moisés Selman

**Affiliations:** 1 Instituto Nacional de Enfermedades Respiratorias Ismael Cosío Villegas, Mexico City, Mexico; 2 Facultad de Ciencias, Universidad Nacional Autónoma de México, Mexico City, Mexico; 3 I. Buendia-Roldan and L. Chavez-Galan contributed equally

## Abstract

A growing body of evidence indicates that a subset of patients with severe COVID-19 may present with persistent lung damage, including fibrotic changes on high-resolution computed tomography (HRCT) and decline of pulmonary function [1–4]. However, studies dealing with biomarkers that may predict an individual's risk of presenting residual lung abnormalities are few.

## Shareable abstract

**This study indicates that increased MMP-7 in patients with post-COVID-19 is associated with lung abnormalities in gas exchange and subtle structural alterations in HRCT suggestive of fibrosis.** https://bit.ly/4etsvk5

*To the Editor:*

A growing body of evidence indicates that a subset of patients with severe COVID-19 may present with persistent lung damage, including fibrotic changes on high-resolution computed tomography (HRCT) and decline of pulmonary function [1–4]. However, studies dealing with biomarkers that may predict an individual's risk of presenting residual lung abnormalities are few.

Matrix metalloproteases (MMPs) are the principal effectors of extracellular matrix degradation but also shed cell membrane proteins and process diverse bioactive mediators, playing a critical role in physiological and pathological responses [5, 6].

MMP-7 is produced by several cell types and contributes to regulating diverse biological processes. MMP-7 is considered a profibrotic molecule, and reports indicate that is increased in patients with idiopathic pulmonary fibrosis [7] as well as in the serum of other interstitial lung diseases and even in asymptomatic individuals with interstitial lung abnormalities [7–10].

A previous report showed that patients with severe COVID-19 have higher plasma levels of MMP-7 after short-term follow-up (median 9 weeks), displaying an inverse correlation with forced vital capacity (FVC) and diffusing capacity for carbon monoxide (*D*_LCO_) [11].

Closely related with MMP-7 is osteopontin (OPN), a secreted pleiotropic glycoprotein that in lungs with idiopathic pulmonary fibrosis colocalises with MMP-7 in alveolar epithelial cells. Importantly, OPN is cleaved and activated by MMP-7 while MMP-7 is induced by OPN, suggesting a positive feedback mechanism [12].

At present, there are no studies evaluating the interaction between MMP-7 and OPN levels and the presence of residual lung abnormalities at long-time follow-up of post-COVID patients. Therefore, we aimed to assess both molecules in patients during acute COVID and after 1-year follow-up.

Serum samples were obtained to measure MMP-7 from 42 COVID-19 patients under invasive mechanical ventilation (IMV) in our institute (April–July 2020, derivation cohort). A second blood sample was obtained in the same patients at 1-year post-COVID (March–July 2021). An independent group of 22 patients suffering severe COVID-19 requiring IMV was also evaluated at one-year post-COVID as a verification cohort, as well as 22 healthy donors (HD) matched by age and sex. The derivation and verification cohorts (DC and VC) were merged to examine the relationship between MMP-7 levels and clinical, functional and imaging (HRCT) data at one year. In a subgroup of 28 patients and 25 healthy donors, we analysed osteopontin levels during the acute infection and at 1-year post-COVID. Serum was collected and kept at −20°C until use; MMP-7 and OPN were quantified by ELISA (R&D Systems, Minneapolis, USA), following the manufacturer's instructions. The study was approved by the Ethical Committee of the Instituto Nacional de Enfermedades Respiratorias (INER, Protocol C41–20). All participants provided informed consent; the family member responsible for the patient signed the consent at the time of hospitalisation, and the patient later corroborated it.

Clinical electronic records were reviewed to obtain demographic and clinical data during hospitalisation. At 1-year follow-up, all patients from the derivation and verification cohorts were examined by HRCT, and we applied a semiquantitative computed tomography (CT) score as previously described [13]. Briefly, this score was calculated based on the extent of lobar involvement (0: 0% involvement; 1: <5%; 2: 5–25%; 3: 26–50%; 4: 51–75%; 5,>75%; range 0–5). The resulting global CT score was the sum of each lobar score (0 to 25). Pulmonary function tests included simple spirometry, *D*_LCO_ (Easy One Pro-Lab; ndd Medical Technologies), 6-Minute Walk Test, and oxygen saturation (pulse oximeter; NONIN).

For the statistical analysis we used the GraphPad Prism 9 program (GraphPad Software, La Jolla, CA, USA). The Kolmogorov–­Smirnov test was used to assess normality, and the Mann–Whitney U-test for comparison between groups. We defined demographic and clinical data as mean±standard deviation and ELISA data as median±25th–75th interquartile range (IQR); p<0.05 was considered as a statistically significant difference. We analyse the receiver operating characteristic (ROC) curve to determine the sensitivity threshold (Y-axis) and 1-specificity (X-axis) of biomarkers to identify alterations in HRCT suggestive of fibrosis.

In the DC (n=42), the average age was 51±13 years, 60% men, 24% smokers, and BMI 30±5 kg·m^−2^. Diabetes mellitus was the main comorbidity (36%), followed by arterial hypertension (26%). In the VC (n=22), the average age was 61±11 years, 64% men, 36% smokers, and BMI 27±4  kg·m^−2^. Diabetes mellitus and arterial hypertension were also the main comorbidities, 64% and 27%, respectively. No differences were observed regarding the severity of the acute phase (ratio of alveolar oxygen tension to inspiratory oxygen fraction (P_AO_2__/F_IO_2__) 146±69 in the DC *versus* 132±46 in the VC (p=0.3), days of mechanical ventilation (24±13 *versus* 20±8, p=0.1), as well as in inflammatory biomarkers. At baseline, all patients had severe respiratory insufficiency and a marked extent of HRCT lesions, mainly ground-glass attenuation, peripheral consolidations, parenchymal bands, and bronchial dilation.

During acute COVID-19, patients exhibited increased levels of MMP-7 compared with HD (6.7 ng·mL^−1^ IQR 4.6–10.1 *versus* 3.9 IQR 3.2–5.0, p<0.0001), and similar results were found after 1-year post-COVID in both, the derivation cohort (7.9, IQR 4.8–11.2 ng·mL^−1^, p<0.0001), and the verification cohort (8.6 ng·mL^−1^ IQR 4.4–12.6 ng·mL^−1^, p<0.0001) (figure 1a). Interestingly, some patients from the derivation cohort displayed an increase of MMP-7 over time, while others showed a decrease or did not change (figure 1b).

**FIGURE 1:**
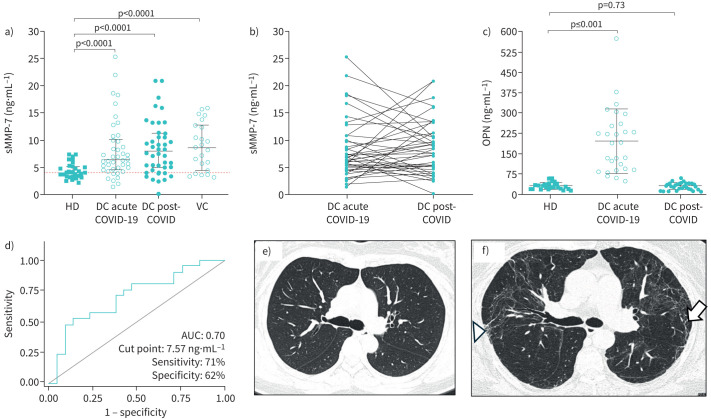
Levels of soluble matrix metalloprotease (MMP)-7 (sMMP-7) in serum of patients with COVID-19. a) High levels of MMP-7 are observed in the acute phase and after one-year post-COVID of the derivation cohort (DC, n=42), as well as in the verification cohort (VC, n=22). Twenty-two healthy donors were evaluated as controls (HD, median showed as red dot line). b) Line plot with individual changes from baseline to 1-year follow-up of the 42 patients of the derivation cohort. c) High levels of osteopontin (OPN) are observed in the acute phase reaching normal levels at one-year post-COVID of the subgroup of derivation cohort (n= 28). d) ROC analysis of MMP-7 to identify their power of discriminate alterations in HRCT suggestive of fibrosis shows an area under a curve 0.70, with a cut point of 7.57 ng·mL^−1^. e) HRCT without pathological changes. f) HRCT from a patient with higher MMP-7 at 1-year post-COVID showing parenchymal bands (arrow) and reticulation (arrow head). Statistical analysis was performed with the Mann–Whitney test; graphs show individual value and median±25th–75th interquartile range; p-value <0.05 was considered significant.

Therefore, to identify whether they had differences regarding lung behaviour, we merged the two post-COVID groups (n=64), and the patients were divided into those with higher or lower MMP-7 values according to the median. Our results showed that patients with higher levels of MMP-7 (n=36) exhibited a lower mean percent predicted *D*_LCO_ compared with patients with lower values (n=28, 85±20 *versus* 75±15; p=0.002), without differences in lung mechanics. Moreover, patients with higher levels of MMP-7 at 1-year follow-up showed a higher CT score (7.6±5.2 *versus* 4.4±3.9; p=0.02) and were more likely to show parenchymal bands, reticulation, and traction bronchiectasis (55% *versus* 29%, respectively, p=0.04) (figure 1f).

ROC curve analysis was conducted to determine the diagnostic value of MMP-7 as a biomarker for identifying alterations in HRCT suggestive of fibrosis. As depicted in figure 1d, the cut-off value for MMP-7 was 7.57 ng·mL^−1^, which yielded 71% sensitivity and 62% specificity, the AUC was 0.70.

Compared with healthy donors, the concentration of oesteopontin showed a significant increase during the acute phase of COVID-19 in both groups (194.7±119.4 ng·mL^−1^ *versus* 29.8±11.4 ng·mL^−1^; p<0.001) and a decrease in the follow-up reaching normal levels at 1-year follow-up (figure 1c).

Our results demonstrated an increased level of MMP-7 in a subset of post-COVID patients after 1-year follow-up, and more importantly, that these patients showed impaired lung diffusing capacity, greater extent of radiological lesions with the CT-score and alterations suggestive of fibrosis.

This finding reinforces the importance of MMP-7, a serum biomarker known to represent lung epithelial injury and extracellular matrix remodelling, as a pathological factor in interstitial lung diseases, likely including persistent lung abnormalities post-COVID. Surprisingly, OPN was elevated during the infection's acute-inflammatory phase, as Hayek S. and colleagues reported [14], but decreased in the follow-up, regardless of residual lung abnormalities. This finding supports the notion that the coincident increase of MMP-7 and osteopontin is particularly associated with idiopathic pulmonary fibrosis as has been previously suggested [15].

Our study has some limitations, including its retrospective design and the sample size. Strengths include a long-term (one-year) follow-up, including a verification cohort within the same period.

In summary, our findings support the notion that increased MMP-7 in patients with post-COVID is associated with lung abnormalities in gas exchange and subtle structural alterations in HRCT indicative of fibrosis suggesting that it may be a potential biomarker. Longer-term observational periods and larger post-COVID cohorts are needed to validate these results and understand this relationship better.
